# Deriving Requirements for Pervasive Well-Being Technology From Work Stress and Intervention Theory: Framework and Case Study

**DOI:** 10.2196/mhealth.5341

**Published:** 2016-07-05

**Authors:** Saskia Koldijk, Wessel Kraaij, Mark A Neerincx

**Affiliations:** ^1^ Radboud University Institute for Computing and Information Sciences Nijmegen Netherlands; ^2^ TNO Netherlands Organisation for Applied Scientific Research The Hague Netherlands; ^3^ Leiden University Leiden Institute of Advanced Computer Science Leiden Netherlands; ^4^ Delft University of Technology Interactive Intelligence Delft Netherlands

**Keywords:** psychological stress, professional burn-out, behavioral symptoms, self-management, health technology, early medical intervention

## Abstract

**Background:**

Stress in office environments is a big concern, often leading to burn-out. New technologies are emerging, such as easily available sensors, contextual reasoning, and electronic coaching (e-coaching) apps. In the Smart Reasoning for Well-being at Home and at Work (SWELL) project, we explore the potential of using such new pervasive technologies to provide support for the self-management of well-being, with a focus on individuals' stress-coping. Ideally, these new pervasive systems should be grounded in existing work stress and intervention theory. However, there is a large diversity of theories and they hardly provide explicit directions for technology design.

**Objective:**

The aim of this paper is to present a comprehensive and concise framework that can be used to design pervasive technologies that support knowledge workers to decrease stress.

**Methods:**

Based on a literature study we identify concepts relevant to well-being at work and select different work stress models to find causes of work stress that can be addressed. From a technical perspective, we then describe how sensors can be used to infer stress and the context in which it appears, and use intervention theory to further specify interventions that can be provided by means of pervasive technology.

**Results:**

The resulting general framework relates several relevant theories: we relate “engagement and burn-out” to “stress”, and describe how relevant aspects can be quantified by means of sensors. We also outline underlying causes of work stress and how these can be addressed with interventions, in particular utilizing new technologies integrating behavioral change theory. Based upon this framework we were able to derive requirements for our case study, the pervasive SWELL system, and we implemented two prototypes. Small-scale user studies proved the value of the derived technology-supported interventions.

**Conclusions:**

The presented framework can be used to systematically develop theory-based technology-supported interventions to address work stress. In the area of pervasive systems for well-being, we identified the following six key research challenges and opportunities: (1) performing multi-disciplinary research, (2) interpreting personal sensor data, (3) relating measurable aspects to burn-out, (4) combining strengths of human and technology, (5) privacy, and (6) ethics.

## Introduction

Employees often report the experience of stress at work, which is related to their well-being. In this research we focus on the population of knowledge workers who are predominantly concerned with interpreting and generating information. Stress is easily caused by their typical working conditions [1]. Several tasks that need to be finished before a deadline, and their course of action is not always self-planned but also determined by external causes, like phone calls, mail, information requests, and other persons or appointments [2]. Following the definition by Selye [3], an employee complaining about stress might mean that his working conditions are very demanding (the stressor), or that he feels that demands put upon him are higher than he can take (the perception of stressors), or that he feels stress reactions in his body such as neck pain or headaches (the experience of stress). To date, the problem of work stress is often approached with questionnaires in which employees are asked to rate various aspects of their work [4,5], followed by department-wide interventions (eg, providing trainings). However, interventions trying to reduce stress have often failed: a recent study with mindfulness at the workplace found no effect on stress levels [6]. Another common approach is finding help with others via group therapy.

As knowledge workers are relatively flexible in their work (when they do what and how they work), there is great potential for them to contribute to the improvement of their own well-being. New technologies are emerging, such as sensors available in mobile phones, smart reasoning, and electronic coaching (e-coaching) apps. In the Smart Reasoning for Well-being at Home and at Work (SWELL) project [7], we see potential in using such new pervasive technologies to address well-being at work at an individual level [8]. We also see possibilities in using unobtrusive and easily available sensors to capture the knowledge workers behavior (eg, computer interactions ,webcam for facial expressions), and infer stress and the context in which it appears. Based upon this information, we aim to develop a system with a suite of support apps that are context aware (ie, optimally adapted to the situation and state of the user). Knowledge workers can then directly act, gaining a more healthy work style, preventing stress building up, and curing stress-related problems like neck pain or headaches. An app could also provide a platform to come into contact with peers for support. Trends like “quantified self” already show the potential of collecting personal sensor data (eg, heart rate, activity patterns) for health improvement. In their paper on technology for well-being, IJsselsteijn et al [9] describe that advancements in sensing and interpretation are promising. They further state that using technology for improving well-being has many advantages including its persistence or objectiveness, the possibility to provide just-in-time notifications with relevant, actionable information or their supportive and motivating role.

**Figure 1:**
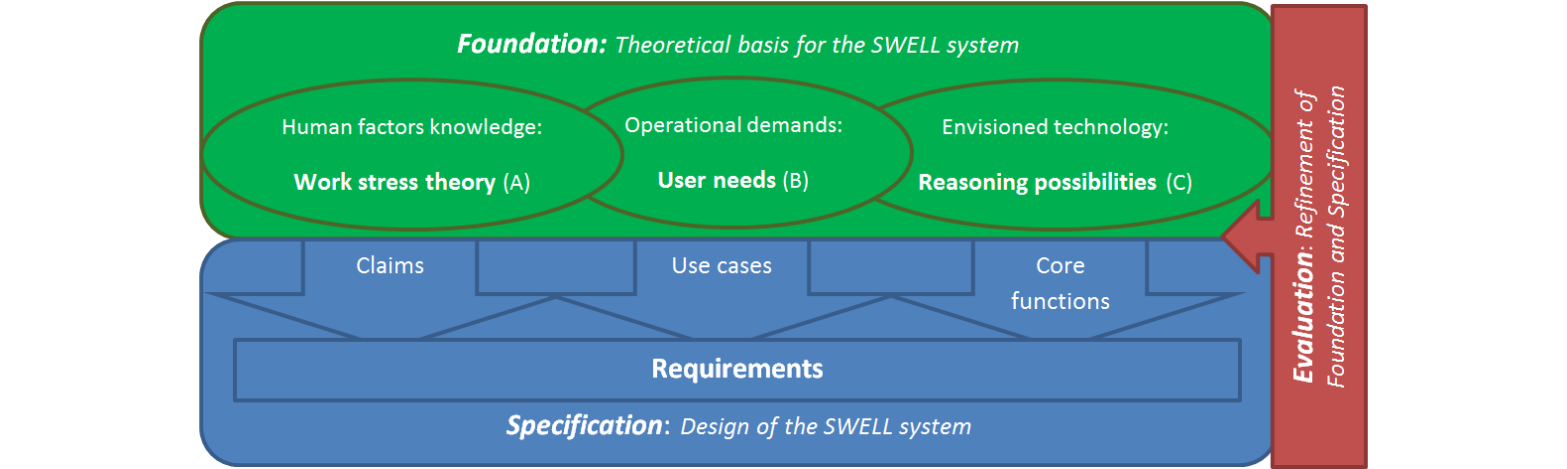
Situated Cognitive Engineering (sCE) approach.

To develop a theoretically and empirically grounded stress self-management system, we take a multi-disciplinary approach. By means of situated cognitive engineering [10], we combine theory on work stress with input on user needs, taking in mind technological possibilities (Figure 1). In this way, a functional system specification with core functions and claims was generated, which is then evaluated with users. The main focus of this paper is the theoretical foundation. The general objective of the SWELL system is to improve well-being at work. An important question is: what defines well-being at work and what causes well-being? Many relevant theories are provided by several disciplines, such as work psychology, biology, or behavioral psychology. However, theories are diverse and different disciplines view the world from different angles (eg, using different levels of abstraction). Therefore, how do different concepts relate to each other? One comprehensive and practical framework that can be used as a theoretical basis for the design of the envisioned self-management support is still lacking. Moreover, psychological theories are often abstract and for implementing a solution many choices need to be made. We investigate the role of new technologies, which also provides new opportunities to study and influence behavior.

**Figure 2:**
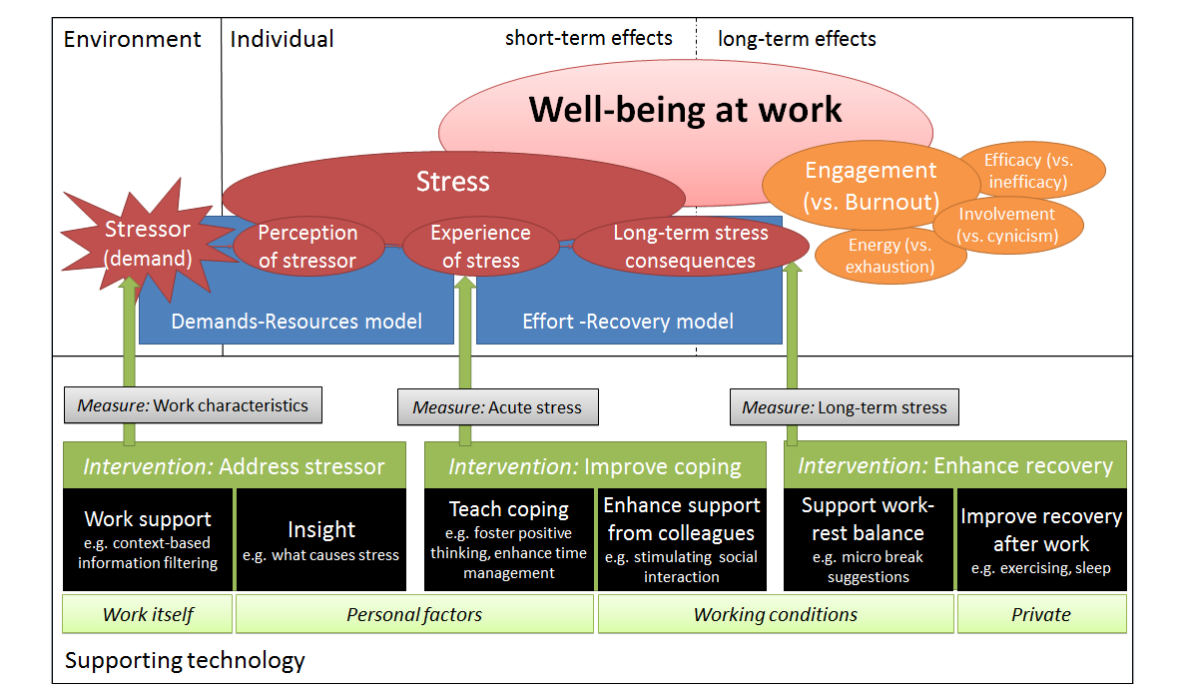
Our framework that combines various stress and intervention theories, as well as possibilities for real-time measurements and interventions with technology.

The main contribution of this paper is a general and pragmatic framework (Figure 2), which combines various stress and intervention theories, as well as possibilities for real-time measurements and interventions with technology. This framework can be used for developing technologies addressing well-being at work, as is demonstrated in our SWELL use case. Moreover, we show that, vice versa, new technologies can also be used for theory building. Our research questions and the remainder of the paper are structured around our framework, beginning with a description of an initial study on user needs as a starting point for the system design. Then, we investigate by means of a literature study, which aspects are relevant to include in the pervasive support system. We answer our *first research question*: which concepts are relevant with respect to well-being at work? The concepts “burn-out”, “engagement”, and “stress” (red/orange parts) are presented. We then use a literature study to investigate which causes of work stress our pervasive system could address. We answer our *second research question*: which person, work, and context conditions can lead to negative stress? We present different work stress models (blue parts). Following that, we integrate knowledge on technical possibilities to define how the pervasive system could quantify relevant concepts (gray parts). We answer our *third research question*: how can sensors be applied to automatically infer stress and the context in which it appears? Finally, we combine insights gained thus far with a literature study on intervention theory (green parts). We answer our *fourth research question*: which interventions can be provided by means of pervasive technology to help a knowledge worker improve his well-being at work? Based on technical possibilities, we define several technology-based interventions (black parts). All parts together form our general framework, which was used to derive requirements for our case study, the pervasive well-being support system SWELL. We present the envisioned system and first prototypes of technical support that were implemented, as well as results from evaluation studies with potential end users. We finish our paper with our conclusions, a discussion of limitations of our work, and a more general reflection, where we present six research challenges that we identified.

## Methods

### Initial Study on User Needs

Following the situated cognitive engineering methodology [10], we start with input from potential end users. We held interviews with five knowledge workers who had experienced burn-out and organized a workshop with seven employees to establish user needs. Knowledge workers indicated that the system should provide them an overview of performed work, preferably in combination with work behavior and the associated subjective experience. This information can then be used by the user to gain insight in work processes. For example, at the end of the day an overview could be provided on how time was spent and how stress evolved. Moreover, users indicated that they would want help in the form of tips. Ideally the tips are also well-timed, taking into account the user’s current context. Finally, users indicated that the system could actively support them during their work. The system can take an active role in supporting the user by filtering irrelevant emails or finding information relevant to the current work topic. We also identified some important factors to address such as not irritating users and addressing privacy. This user input was used to guide the further design of the system. In the next sections we focus on important relevant domain knowledge.

### Well-Being at Work Concepts

In this section we aim to answer our *first research question*: which concepts are relevant with respect to well-being at work? To answer this question, we performed a literature review [11]. The search engine “Web of Science” was used with the keywords well-being, commitment, satisfaction, stress, and engagement. Based on 23 scientific publications an overview of the different concepts was made. The literature review revealed that there are many different related concepts and many different models. Finally, the concepts “engagement” and “stress” were chosen, as they seemed most suitable to capture with sensors. In this section, we first describe the concept of engagement in more detail (Figure 2), and then present literature regarding stress and its consequences.

#### Engagement and Burn-Out

**Figure 3:**
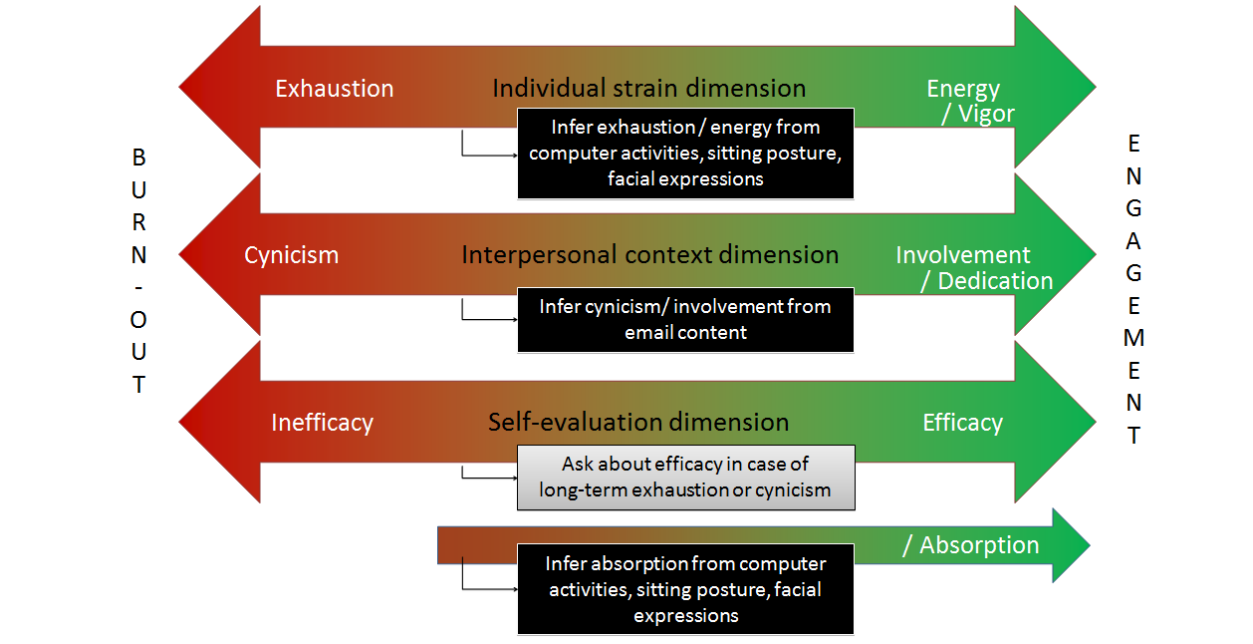
Well-being at work concepts "burn-out" and "engagement" and ideas to infer certain aspects from captured (sensor) data.

The relationship people have with their jobs can be described as a continuum between engagement and burn-out (Figure 3) [12]. Maslach and Leiter distinguish the following three dimensions: (1) individual strain (exhaustion vs energy), (2) interpersonal context (cynicism vs involvement), and (3) self-evaluation (inefficacy vs efficacy). According to this terminology, an engaged employee feels energy, involvement, and efficacy. His state can be characterized as worrisome when he feels exhaustion, cynicism and/or inefficacy, which characterizes burn-out [13]. According to Maslach and Leiter [12], “engagement represents a desired goal for any burn-out interventions.” (p 499) Schaufeli and colleagues describe engagement as the combination of vigor, dedication, and absorption [14]. The first two concepts are similar to those described by Maslach and Leiter [12]; however, the main difference lies in the third dimension, absorption, which is not the opposite of inefficacy, but a different aspect.

#### Stress

Besides engagement or burn-out, a relevant concept that can be experienced in the office is stress. In research we find that the term stress is often used to refer to different things. In our work we use the definition by Selye (Figure 2) [3]. An environmental demand, or stressor, leads to a perception of the stressor, which is dependent on the particular characteristics of the individual. The individual’s perception of the stressor results in a particular experience of stress. An employee complaining about stress might thus mean that his working conditions are very demanding (the stressor), or that he feels that demands put upon him are higher than he can take (the perception of stressors), or that he feels stress reactions in his body (the experience of stress).

Selye distinguishes good stress (eustress) and bad stress (distress) [15]. Some amount of stress is not harmful and might even be beneficial to gain concentration and focus. Eustress occurs when the person experiences the right amount of demand. Distress occurs when a person experiences too much or too little demand. This is related to the Yerkes Dodson Law [16], which describes that (empirically) performance improves with arousal, up to a certain point, after which it declines again.

Individual characteristics and appraisal play an important role in the experience of stress. The same stressor can be seen as a problem leading to negative emotions causing distress, or as challenge leading to positive emotions causing eustress [17]. This can depend on the amount of resources or feeling of control that the individual has. So even changing the mind-set of a knowledge worker could help him cope better with stressors. More details on the balance of demands and personal resources can be found in the section on work stress models.

**Figure 4:**
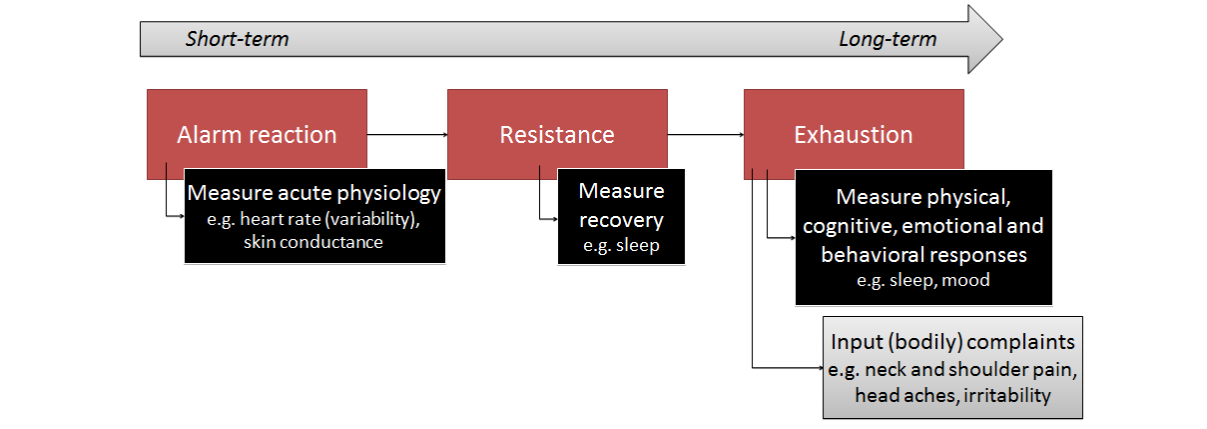
Stress reactions of the body and measuring possibilities.

The body’s short- and long-term reactions to stress can, from a biological perspective, be captured in the following three stages and in Figure 4 (General Adaptation Syndrome [18]): (1) alarm reaction - the fight or flight response, (2) resistance - the body adapts to the stressor, and (3) exhaustion - the body's resistance decreases due to long-term stress. The alarm reaction causes adrenaline to spread through the body and a rise in blood pressure (reaction of the nervous system). Under very stressful conditions, a shift in hormone production may take place, increasing stress hormones like cortisol, which increases blood sugar, but also suppresses the immune system (reaction of the hormonal system). This stress response system works well for dealing with short-term stressors. When the stressor disappears the body gains back its natural balance. When the level of the stress hormone cortisol is high for a prolonged time, negative effects on the brain, for example, can occur. This shows the importance of recovery.

With lack of recovery, stress can accumulate and lead to health problems. Extended periods of stress can cause physical reactions (eg, increased blood pressure, muscle tension, headache, sleeping problems), cognitive reactions (eg, problems with concentrating, problems with setting priorities, decreased efficiency in work), emotions (eg, irritation, feeling restless, tense, anxious), and changes in behavior (eg, avoiding social contact, more risk taking, not being able to relax, increased complaining) [19]. Moreover, Bakker et al [20] explain that stress can not only directly lead to illness through its physiological effects, but also indirectly through maladaptive health behavior such as smoking, poor eating habits, or lack of sleep.

#### Relevant Concepts for the System

In this section we aimed to answer our *first research question*: which concepts are relevant with respect to well-being at work? We identified the concepts stress and engagement (vs burn-out), with three underlying dimensions: energy, involvement, and efficacy (or absorption) (Figure 2, orange/red parts). Moreover, we found that stress is a normal process and in the form of eustress also good for well-being and performance. It cannot be the goal to prevent stress. Rather, employees should be helped to handle distress and prevent negative long-term consequences. In a pervasive system, we could measure the stressor itself (eg, work characteristics), as well as the individual’s perception of the stressor (eg, acute stress). In addition, we could analyze long-term patterns in which stress is building up and measure recovery (eg, sleep time or the amount of physical activity).

#### Core Functions of the System

Based upon this part of the theoretical framework, we formulated the core functions (F) for the pervasive well-being system: the SWELL system could collect information about aspects of engagement, work characteristics, acute stress, and long-term stress and recovery (F1). Its associated claim states that this information is useful for data-driven and context-aware coaching.

### Causes of Work Stress

**Figure 5:**
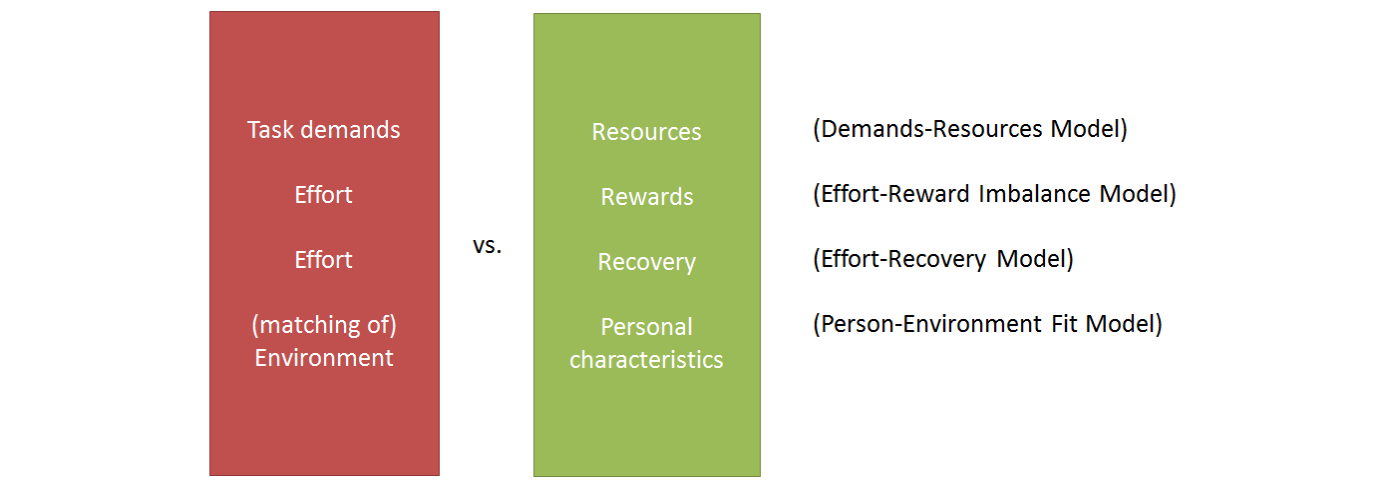
Different work stress models.

After having described the concepts related to well-being at work, we now turn to models describing underlying causes. We aim to answer our *second research question*: which person, work, and context conditions can lead to negative stress? We present the four most influential work stress models, which all describe a balance between two variables (Figure 5). The basic idea is that work becomes stressful when high demands are combined with (1) insufficient resources (such as low job control and little social support); (2) little rewards; (3) little recovery; or (4) an environment that mismatches with personal characteristics. We now outline each model in more detail. Based on each model, we identify aspects that can be addressed by means of technology.

#### Job Demands-Resources Model

**Figure 6:**
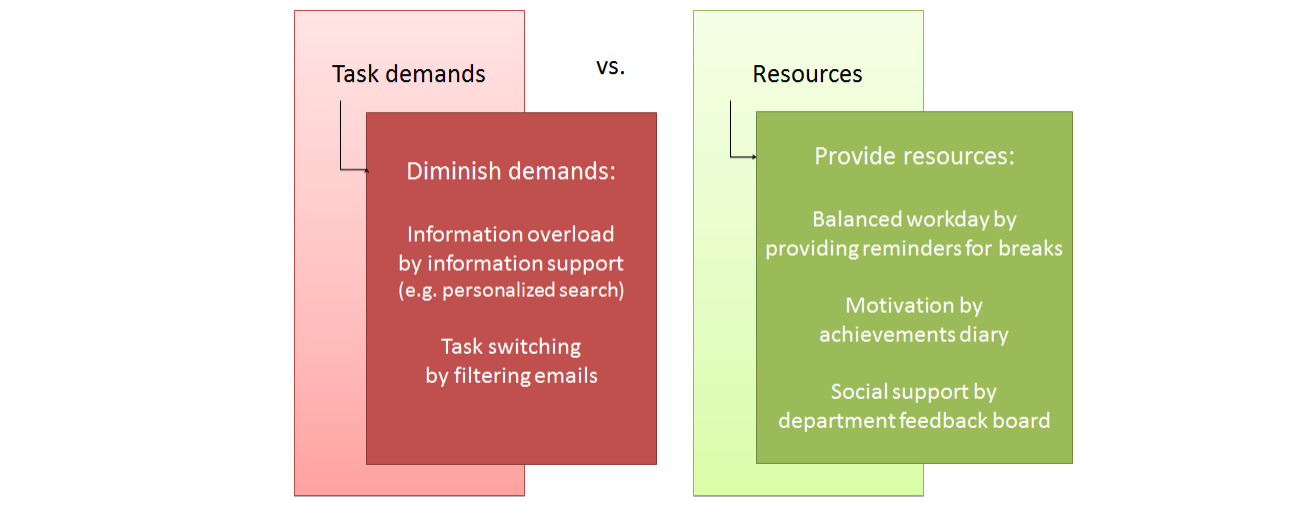
Job Demands-Resources model [12], and possibilities for technological support.

The first model can be characterized by a balance between job demands on the one hand and resources on the other hand (see Figure 6). Karasek Jr developed the initial model called the Job Demands Control (JDC) model [21]. The model was later extended to the Job Demands-Resources (JD-R) model [22]. Here, the more general interplay between job demands and job resources is described. Job demands are aspects of the job that require effort (eg, physical workload, time pressure, emotional demands, the physical environment). High job demands are not problematic; problems arise when the necessary resources lack. Job resources are aspects of the job that help in achieving work goals, reduce demands, or stimulate personal growth and development such as autonomy, job control, social support (from colleagues, supervisor, family, and peer groups), feedback, rewards, task variety, and role clarity. The WEB (Werkstressoren-Energiebronnen-Burnoutmodel) model [23] is another variant of the JD-R model, in which a direct link between demands, resources, and three aspects of burn-out is made. The aspects of burn-out are high demands cause exhaustion, whereas a lack of resources can lead to a decreased feeling of competence (inefficacy), and distancing oneself from work (cynicism).

Based on the JD-R model, we can address well-being at work from two sides. First of all, we can diminish the demands placed upon knowledge workers: a typical demand on a knowledge worker is to deal with large amounts of information. We can make technology that can try to diminish information overload by providing information support, for example, in the form of filtering context-relevant from irrelevant emails (Technology T01) or by enabling personalized search (T02). Another demanding aspect of the work is task switching. A computer tool could diminish this demand by helping employees to remain focused on the task at hand by filtering irrelevant emails (T01 again) or with gamification, motivating employees to stay focused by giving points for less task switching (T03).

Secondly, we can provide additional resources. A resource that the knowledge worker has is his motivation and self-efficacy. The computer tool can support motivation by providing an achievements diary (T04), which is in line with work by Amabile and Kramer [24], who showed that the feeling of making progress leads to more motivation and better performance. We could also facilitate social support, facilitate support by peers by use of a department-wide feedback board (T05). Another resource is a good work-rest balance with variation in tasks. The system could help to have a balanced workday by providing insights in what gives and costs energy by providing an activity and workload overview and promoting better planning (T06). Taking enough recovery breaks could also be traced and supported with technology (T07). It is imperative to consider keeping the knowledge worker in control and not posing additional demands.

#### Effort-Reward Imbalance Model

The Effort-Reward Imbalance (ERI) model [25] can be characterized as a balance between effort on the one side and rewards on the other side. As long as the rewards are in balance with the efforts of the employee there is no problem. An imbalance might occur when the employee’s efforts are higher than his rewards, which might happen due to over-commitment. Such an imbalance may result in stress and negative consequences for health.

Based upon the ERI model, we can address well-being at work by helping employees to match their efforts to the expected rewards. We might support realistic goal setting and in this way diminish pressure and disappointments. Insight regarding planned time versus the real-time may facilitate better (re)planning and setting more realistic goals (T06 again). Moreover, looking back at ones achievements could help employees to get a better feeling of their productivity (T04 again). Also aspects of gamification might provide employees small motivating rewards, for example, collecting points for staying focused (T03 again).

#### Effort-Recovery Model

**Figure 7:**
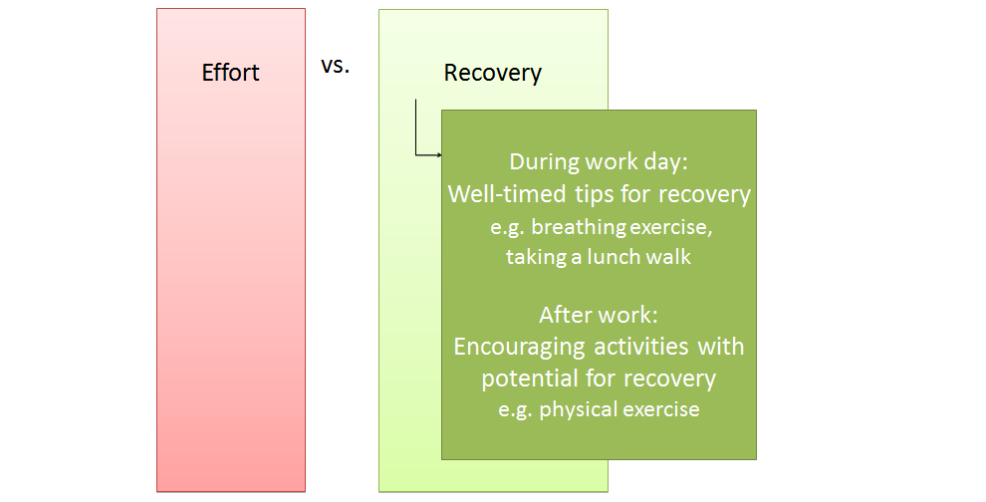
Effort-Recovery model [33], and possibilities for technological support.

The Effort-Recovery (E-R) model [26] can be characterized as a balance between effort and recovery (Figure 7). Here, Meijman et al describe that job demands and resources lead to negative strain during work. After work, home demands and resources lead to strain reactions. The individual can perform activities which can have a positive effect on recovery, leading to a particular psychological and energetic state at bedtime. By means of sleep, additional recovery can be gained and the individual starts the next workday with a certain psychological and energetic state. Failing to recover enough from strain can make the experience of work demands the next day higher and the experienced resources lower, leading to even more strain. This process can be a vicious circle. According to Demerouti and colleagues [27] lack of recovery can “result in an accumulative process developing into chronic load reactions or allostatic load according to McEwen’s (1998) allostatic load theory, such as chronically elevated heart rate, hypertension, chronic fatigue, and persistent sleep problems.”

Four important dimensions play a role in recovery [28]: psychological detachment, relaxation, mastery, and control. Psychological detachment from work can bring the psychophysical system back to its normal state. Relaxation causes decrease in physical activation. Controlling what activity to perform can improve esteem and efficacy. Mastery in performing challenging activities can cause improvement of skills, competence, and esteem.

In general, physical activity seems to be a good means for recovery [29]. Research showed that “in an adolescent population aerobic training does appear to provide some benefits with regard to psychological stress and well-being.” Hassmen et al [30] found that “individuals who exercised at least two to three times a week experienced significantly less depression, anger, cynical distrust, and stress than those exercising less frequently or not at all.”

Based upon the E-R model, we can address well-being at work by making employees aware that recovery during work and non-work time is very important. Interventions could be aimed at taking well-timed breaks during the work day (again T07): passive, as well as active breaks, could be suggested such as relaxation or taking a lunch walk. On the other side, an important aspect of improving well-being at work is also what someone does in his free time. We see that activities after work give potential for recovery. This model is interesting within the SWELL project, as it can combine the domains of well-being at work and at home. Interventions for more well-being could be aimed at better relaxation or detaching from work by means of a hobby (T08), for example. Addressing physical fitness could also be a good intervention (T09).

#### Person-Environment Fit Model

The Person-Environment (P-E) fit model describes a fit between person and environment characteristics. A misfit between the person and his environment can lead to strain, with the danger of illness. There can be a misfit between personal abilities and environmental demands or between personal needs and environmental supplies [31,32]. Leiter and Maslach [33] developed the Areas of Worklife Scale (AWS) around this idea. They say that “the greater the perceived gap between the person and the job, the greater the likelihood of burn-out; conversely, the greater the consistency, the greater the likelihood of engagement with work.” The AWS has items on six aspects: workload, control, reward, community, fairness, and values.

Based upon the P-E fit model, we can address well-being at work by helping employees realize that performing tasks that fit their personal preference is very important for their well-being. Tasks that give energy and tasks that cost energy could be identified by providing an overview over tasks and energy levels over the day (again T06). In the future, the employee can then try to find work fitting his preferences more.

#### Addressing Causes of Work Stress

In this section we aimed to answer our *second research question*: which personal, work, and context conditions can lead to negative stress? We elaborated on several work stress models that describe how stress in working environments is caused. The different models all have a different focus and complement each other. There are no specific personal, work, or context conditions that generally lead to stress. Work becomes stressful when high demands are combined with (1) insufficient resources; (2) little rewards; (3) little recovery; or (4) an environment that mismatches with personal characteristics. The most useful models for developing pervasive systems are the JD-R model and the E-R model, which we integrated into our framework (see Figure 2, blue parts). The JD-R model describes how (environmental) stressors can cause the experience of stress. The E-R model describes how the experience of stress can lead to long-term stress consequences. We presented several ideas on how technology can diminish demands, enhance resources, or help with recovery. An overview of identified technologies, the underlying models, and the associated claims are provided in Table 1.

**Table 1:** Overview of identified technologies and associated claims.

ID	Possibility for technological support	Underlying theory	Claim
T01	Filtering emails	JD-R model	Diminishes demands by reducing information overload
T02	Personalized search	JD-R model	Diminishes demands by reducing information overload
T03	Gamification facilitating focus	JD-R and ERI models	Diminishes demands by diminishing fragmentation, enhances motivation by means of small rewards
T04	Achievements diary	JD-R and ERI models	Enhances resources or rewards by fostering motivation
T05	Department-wide feedback board for peer support	JD-R model	Enhances resources by means of social support
T06	Activity and workload overview for insight	JD-R, ERI, P-E Fit models	Provides insight in the balance between demands and resources, efforts and rewards, or person-environment fit
T07	E-coach for taking enough recovery breaks	JD-R and ERI models	Enhances resources or recovery by taking rest breaks
T08	E-coach for relaxation or detaching after work	E-R model	Enhances recovery by detaching
T09	E-coach addressing physical fitness	E-R model	Enhances recovery by releasing stress with physical activity

Note that all models describe work stress in qualitative terms. Our aim is to quantify several aspects by using sensors. For example, demands could be quantified by measuring work characteristics (eg, tasks and content worked on), personal resources could be quantified by measuring the associated acute stress (eg, physiological stress responses, mental effort), and recovery of the individual could be quantified by measuring long-term stress aspects (eg, sleep time, physical activity).

## Results

### Inferring Stress and its Context

After having described concepts related to well-being at work and causes of work stress, we now focus on assessing stress and its context. In current practices, most often questionnaires are being used [4,5]. However, this data collection has several shortcomings since data is self-reported, it can suffer from recall bias and subjectivity, and data is only collected once a year, for example. Using sensors overcomes these shortcomings because can be collected in an objective way, in real-time, and in an office context. Such data about stress, together with the context in which it appears can give insights that can more directly be acted upon by an employee.

Therefore, we now aim to answer our *third research question*: how can sensors be applied to automatically infer stress and the context in which it appears? We focus on (physically) unobtrusive, relatively cheap sensors that can easily be used in office environments. Following the situated cognitive engineering methodology [10], we integrate knowledge on technical possibilities. We also investigate user choices regarding data collection.

#### Technical Possibilities

**Figure 8:**
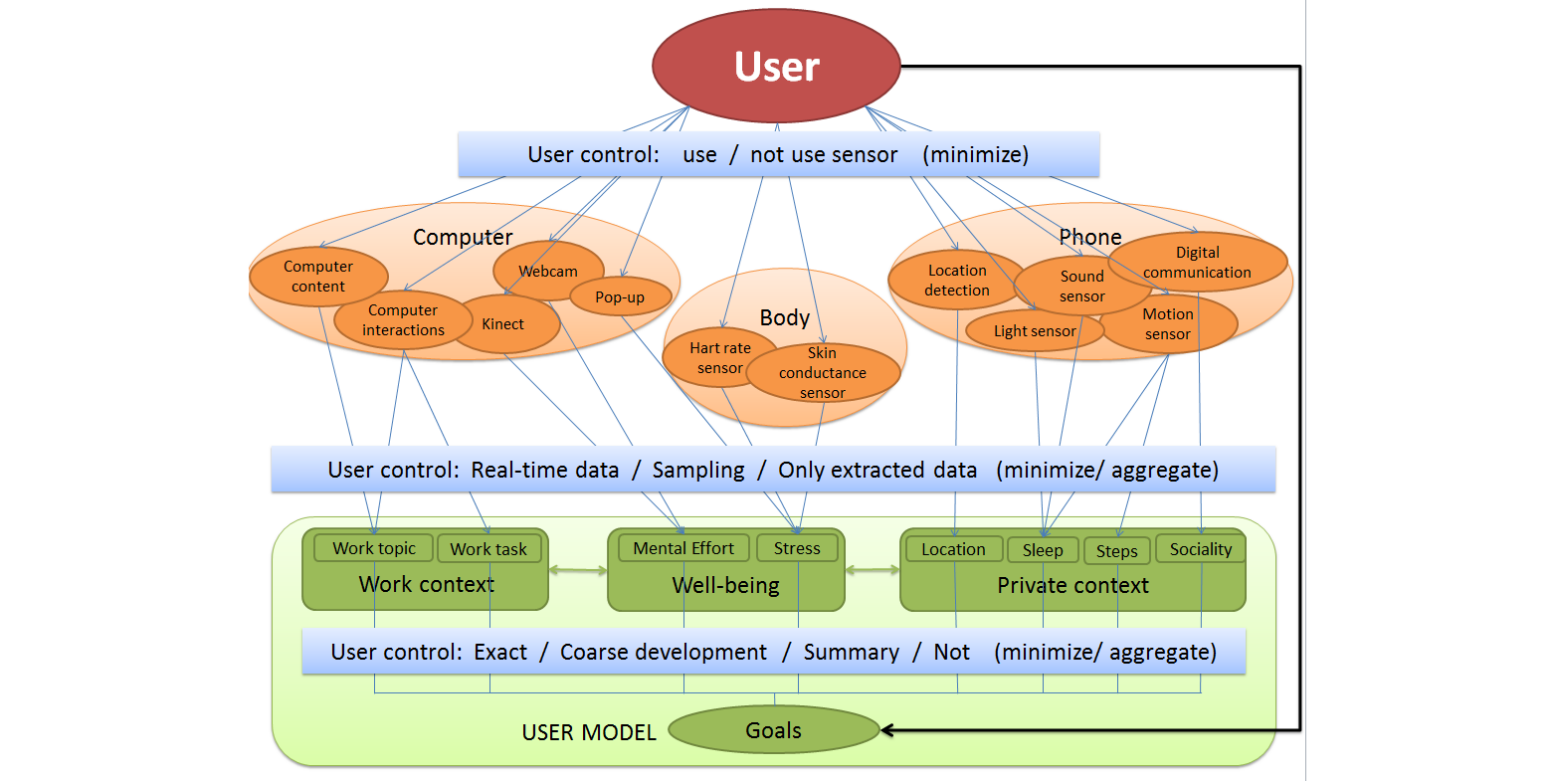
Overview of the system and its user's model, which holds information on the users work context and well-being.

In the previous sections, we identified several relevant concepts that the system could measure to provide data-driven coaching and context-aware support: work characteristics, acute stress, long-term stress and recovery, and aspects of engagement. An overview of the types of information and the sensors that can be used in the pervasive system to infer these aspects is presented in Figure 8.

##### Work Characteristics

First of all, we can measure work characteristics. The task (eg, write report, search information) someone is performing can be inferred from computer interaction data. We present algorithms for real-time task inference [34]. Moreover, which project someone is working on can be detected by analyzing the content of accessed documents and websites. We also present algorithms for topic detection [35]. The combination of tasks and topics can provide valuable information on the context in which stress appears. Based upon information on what someone was working on, we can also infer the amount of task switching, variation in tasks, and the work-rest-balance. Most informative are probably deviations from usual behavior of the specific user.

##### Acute Stress

With respect to inferring of stress from sensor data, Sharma and Gedeon [36] provide a compact survey. Often, body sensors are used to measure the physiological stress response directly, for example skin conductance [20] or heart rate [37]. More and more unobtrusive devices are entering the market, like measuring watches, so this might be a potentially interesting measure to use. As a critical side note, however, these devices may not be accurate enough to determine the more insightful variable of heart rate variability (HRV). Moreover, many external influences on physiology exist (eg, drinking coffee or physical activity). Asking the user himself for input on stress may be useful.

There is also potential in using outward characteristics, such as facial expressions, postures, or computer interactions as indicators for the user’s mental state. Facial expressions are currently mainly used for inferring emotions, but facial expressions could also show cues to infer other mental states that might be more relevant in a working context. In earlier work, where working conditions were manipulated with stressors, we found that specific facial action units may be indicative of experienced mental effort [38]. Research by Dinges et al [39] suggests that facial activity in mouth and eyebrow regions could be used to detect stress. Moreover, Craig and colleagues [40] looked at facial expressions while students worked with an online tutoring system. Association rule mining identified that frustration and confusion were associated with specific facial activity. Mental states are also being estimated from computer interaction data. Results by Vizer et al [41] indicate that stress can produce changes in typing patterns. Finally, Kapoor and Picard [42] describe work on recognizing interest in students by means of computer interactions and postures. Currently, we are also investigating how far we can infer stress or experienced mental effort from facial expressions, computer interactions, and postures [38]. Due to individual differences, general models will have to be adapted to the specific user for reliable estimates.

##### Long-Term Stress and Recovery

To measure the more long-term physical, cognitive, emotional, and behavioral responses, as well as recovery from stress (Figure 4), it may be interesting to include aspects of the private context, outside work. With mobile phone sensors, a rough estimate of sleep time can be provided by the combination of darkness, silence, and recharging of the phone battery [43]. Moreover, the amount of physical exercise, which is a good relief for stress, can be measured by means of sensors (eg, via mobile phone [43], band [44]). A very rough estimate of sociality can be made based upon the amount of phone communication. In addition, location information, such as that obtained by Global Positioning System (GPS) can be useful to enhance the timing of feedback.

##### Aspects of Engagement

Besides the aspects already included in Figure 8, we have some initial ideas to measure certain aspects of engagement (Figure 3) during work. Based on sensor data, energy (vs exhaustion) may be a concept that can be inferred by looking at someone's sitting posture, computer interactions, or facial expressions. This could give longitudinal information on the individual strain of an employee. Moreover, we could get a first indication of involvement (vs cynicism) from textual analyses of email content. A state of absorption, like “flow”, might be recognizable based on computer behavior (eg, focus on one application), typical postures (eg, leaning forward, sitting still), or facial expressions. The concept of efficacy (vs inefficacy), however, might probably best be assessed with questions to the knowledge worker. For example, when the longitudinal data shows little energy, the employee might want to fill in some questions on feelings about his efficacy, to be able to give an early warning and provide help in time.

#### User Choices Regarding Data Collection

To estimate the identified states, various sensors are necessary (Figure 8). Applying sensor technology to monitor personal activities most probably raises concerns related to privacy. Therefore, we performed a user study to investigate what the general perception of using various types of information and sensors is.

Nine participants tested a sensing and e-coaching prototype for two weeks. In a questionnaire, they were then asked to set the configurations for data collection to be used for their own insight and for improving the e-coaching app. We found that some sensors are, in general, perceived as more privacy sensitive (eg, webcam, sound sensor, computer content, and digital communication), others as less privacy sensitive (eg, motion sensors, heart rate, and skin conductance). However, preferences regarding data collection are diverse and depend on the goal for which they want to use the system and the trade-offs they make for themselves regarding privacy. The system should therefore be configurable, such that the user can (1) decide which sensors to use; (2) decide in which detail information is extracted from the sensors; and (3) decide to store information in exact or only aggregated form (Figure 8). Users may want to experiment with how much functionality they can gain with disclosing certain types of data.

#### Using Sensing and Reasoning

In this section we aimed to answer our *third research question*: how can sensors be applied to automatically infer stress and the context in which it appears? We provide an overview of all possibilities for real-time measurements in Table 2. The user study showed that user’s are only interested to collect data that is necessary for supporting their specific goal, so the system should be configurable.

**Table 2:** Overview of the three aspects in the stress chain (from left to right). For each aspect, several indicative factors can be measured and different technology-based interventions can be provided .

Problem	Stressors: “My environment poses high demands”	Experience of stress: “I feel I cannot handle all demands”	Stress consequences: “I experience stress symptoms”
Measure	Work characteristics	Acute stress	Long-term stress and recovery
Concept and how to infer	Tasks and content worked on: computer activity	Physiological stress responses: skin conductance and heart rate (variability) from measuring watch	Sleep time: mobile phone sensing, using the combination of silence, darkness and recharging of the phone battery
	Variation in tasks, task switching, work-rest balance: computer activity (also calendar)	Mental effort: infer from facial expressions, posture, computer activity	Physical activity: accelerometer, GPS
Intervention	Address stressors (primary prevention)	Enhance coping (secondary prevention)	Enhance recovery (tertiary prevention)
Example technology	Providing work support: filtering emails (T01) and personalized search (T02)	Helping to improve coping abilities: gamification for focus (T03) and achievements diary (T04)	Supporting work-rest balance: e-coach for recovery breaks (T07)
	Providing insight in the sources of stress: activity and workload overview (T06)	Fostering support by colleagues: department-wide feedback for peer support (T05)	Helping to improve recovery after work: e-coach for detaching after work (T08) and e-coach for physical fitness (T09)

#### Core Functions of the System

The identified core functions of the system, together with the associated claims are shown in Textbox 1.

**Core function of the system and the associated claims.**

Core functions and claims

• F1.1: The SWELL system shall infer relevant information from unobtrusive sensors to provide real-time objective measurements.

• Claim: Sensors provide real-time information on stress and the context in which it appears, which the employee can directly act upon.

• F1.2: The SWELL system shall only collect data that is necessary to support the user’s goal.

• Claim: User’s are only willing to collect information relevant to their personal goal (due to privacy).

### Improving Well-Being at Work

We have described concepts related to well-being at work, causes that play a role in the experience of stress, and means to assess relevant aspects with sensors. As a next step we aim to find an answer to our *fourth research question*: which interventions can be provided by means of pervasive technology to help a knowledge worker improve his well-being at work? We describe intervention and behavioral change theory.

#### Intervention Theory

There are different possibilities to address well-being at work and diminish stress. First of all, one can distinguish prevention approaches aimed at *different stages* in the stress chain (Figure 2; upper green parts) [45]. Primary prevention is aimed at the stressors, such as changing the work or work situation to prevent risks. Secondary prevention is aimed at the (short-term) stress reactions, including helping employees to develop good coping strategies to handle stress risks and their consequences. Tertiary prevention is aimed at addressing (long-term) stress consequences, such as promoting a balanced life style to recover.

Moreover, interventions can target different areas (Figure 2; lower green parts). Based on the literature, we identified four areas: the work itself, personal factors, the working conditions, and private circumstances [11]. To support the employee to reach more well-being, the intervention should be targeted at the problem area. First of all, one could change the work itself, improve work planning, or get a more focused work-flow. Secondly, the intervention can target personal factors. One could enhance self-knowledge (eg, what causes my stress), or improve active coping. Fourth, the intervention can target working conditions. One can address organizational aspects, social aspects (eg, support from colleagues), or the work-rest balance. Finally, the intervention can address private circumstances. One can address social aspects (eg, support from friends), or recovery.

Finally, we can distinguish *various types* of stress reducing interventions [46]. The most suitable type of intervention may depend on the employee’s preference: cognitive-behavioral (eg, coping skills and being more assertive), creativity, exercise, food, journaling, relaxation, social, or time-management and organizational. Note that an intervention can be social and creative at the same time.

#### Behavioral Change

Until now, we explained what aspects interventions may address improvement in well-being at work. However, changing the behavior of an individual may be difficult, especially in the context of (bad) habits. Therefore, we now consider behavioral change theory [47].

People may know that particular behavior may be good for them, but still they may sustain their old behavior. Fogg [48] identified three main hurdles preventing humans to perform the right or healthy behavior: lack of ability, lack of motivation, and lack of a well-timed trigger. The interventions should be designed in a way that they address these hurdles. More specific relevant determinants to address are risk awareness, motivation, social influences, skills, self-efficacy, supportive environment, attention, and behavioral awareness.

**Figure 9:**
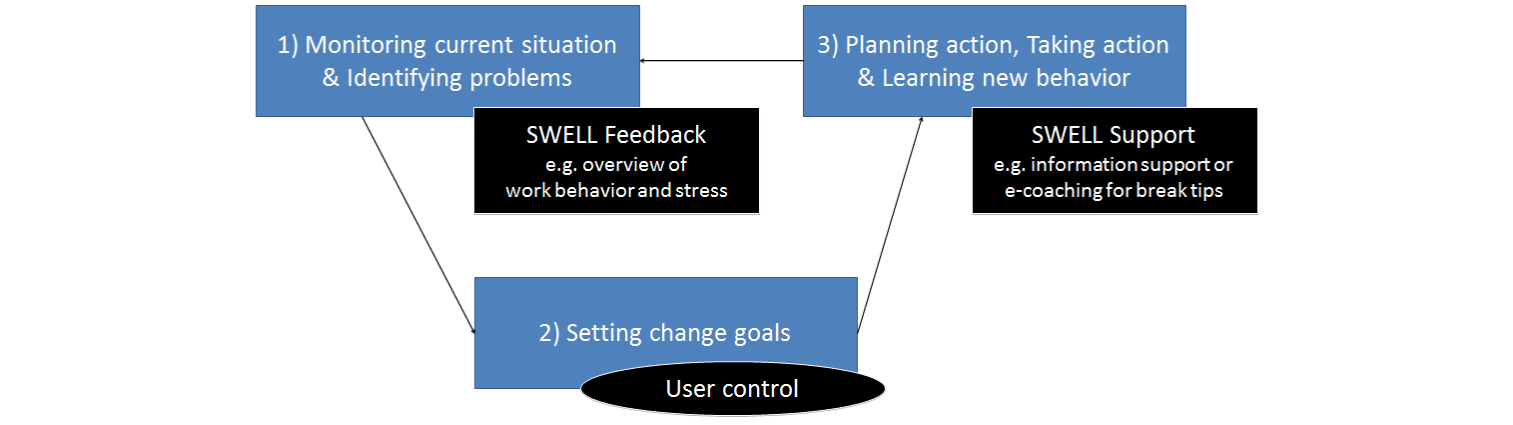
Behavior change and how technology could support it.

For someone to successfully change his behavior, the following three main aspects should be supported in the system (Figure 9): (1) monitoring current situation and identifying problems, (2) setting change goals and planning action, and (3) taking action and learning new behavior.

We identified the most appropriate behavior change techniques [49] for the pervasive system, based on the list presented by de Korte et al [50], and they include feedback, self-monitoring, contextual risk communication, and reminders or cues to action.

#### Technology-Based Interventions

In this section we aimed to answer our *fourth research question*: which interventions can be provided by means of pervasive technology to help a knowledge worker improve his well-being at work? The system can address the stressor, improving coping, and enhancing recovery in the stress chain (Figure 2). In addition, we address the work itself, personal factors, working conditions, or private aspects.

**Figure 10:**
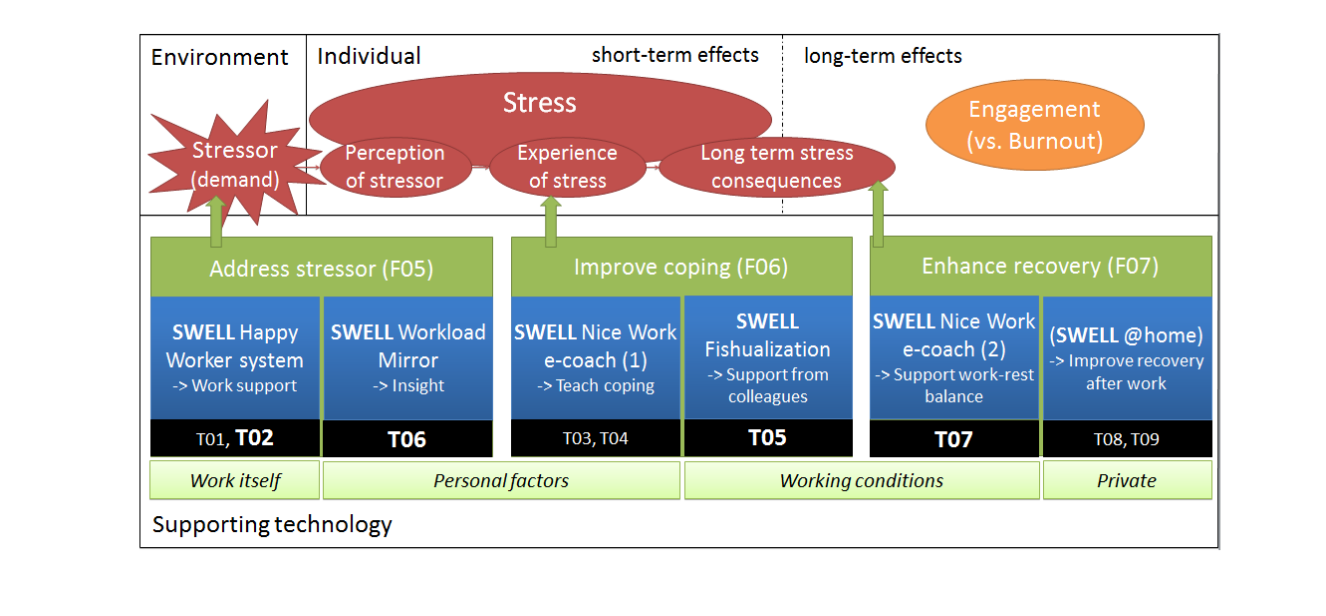
SWELL system functionality in our general framework.

Finally, the pervasive system should also support the employee throughout the behavioral change chain, and specifically address barriers towards changing behavior. We show how the specific supporting technologies identified in the section on work stress models can be placed into this framework (Figure 10). Further technology-supported interventions can be designed based upon our framework, and some ideas are included in Figure 2 (black parts).

#### Core Functions of the System

The identified core functions of the system based upon this part of the theoretical framework and the associated claims are shown in Textbox 2.

**Identified core functions of the system and the associated claims.**

Core functions and claims

- F2: The SWELL system shall address three different causes of stress: address the stressor (F2.1), coping (F2.2), and recovery (F2.3).
- Claim: By providing different types of interventions, different causes of stress can be addressed with the system, making it usable in more situations.
- F3: The SWELL system shall foster behavioral change by helping to monitor the current situation and identifying problems (F3.1), letting the user set personal goals and enable specific functionality (F3.2), and helping to learn new behavior by fostering the ability, motivation or trigger to take action (F3.3).
- Claim: By using behavior change theory the system will be more effective in actually bringing about behavioral change regarding well-being at work.

### Envisioned System and Evaluation of Prototypes

The formulated core functions for the system are summarized here. The envisioned pervasive SWELL system supports the knowledge worker to improve well-being at work (OBJ). The SWELL system could collect information about aspects of engagement, work characteristics, acute stress, and long-term stress and recovery (F1). The SWELL system shall infer relevant information from unobtrusive sensors to provide real-time objective measurements (F1.1). The system only collects data that is necessary to support the user’s goal (F1.2). With respect to behavioral change, the user will start with getting insight in his situation and identifying problems that he wants to address (F3.1). Based on these insights the user can then set personal goals and enable specific desired SWELL functionality (F3.2). In case the environment poses high demands, the user may decide to address some of his stressors (F2.1). In case the user feels overwhelmed by demands placed upon him, he may decide to address some of his coping abilities (F2.2). In case the employee experiences stress symptoms, he may decide to enhance recovery (F2.3). Behavior change techniques are used to foster motivation, ability and triggers to take action (F3.3).

We built the first prototypes of different SWELL functionality and show how the prototypes fall into our framework in Figure 10. All of the systems are aimed at improving well-being at work and most prototypes make use of sensor information (Textbox 3).

**The different SWELL functionalities.**

SWELL prototype systems

- The SWELL Workload Mirror is an implementation of T06 “activity and workload overview” and provides insights regarding stress and the context in which it appears. It tries to tackle stress in the beginning of the stress chain (eg, what causes stress?) with the aim of helping employees to address the stressor itself. Based on these insights, the user might want to use one of the other SWELL systems for support.
- The SWELL HappyWorker system is an implementation of T02 “personalized search” and helps employees find relevant information. It tries to tackle stress in the beginning of the stress chain (diminishing demands) with the aim of addressing the stressor itself.
- The SWELL Fishualization is an implementation of T05 “department-wide feedback for peer support” and is aimed at fostering awareness and communication about stress at work. It tries to tackle stress in the middle of the stress chain, helping employees to cope with stress.
- The SWELL NiceWork app is an implementation of T07 “e-coach for recovery breaks” and provides interventions aimed at improving coping, and enhancing recovery. It tries to tackle stress in the middle and end of the stress chain.

We now describe two of the prototypes, the SWELL Fishualization and SWELL NiceWork apps, in more detail, together with their first small-scale user studies.

#### Fostering Colleague Support - SWELL Fishualization

**Figure 11:**
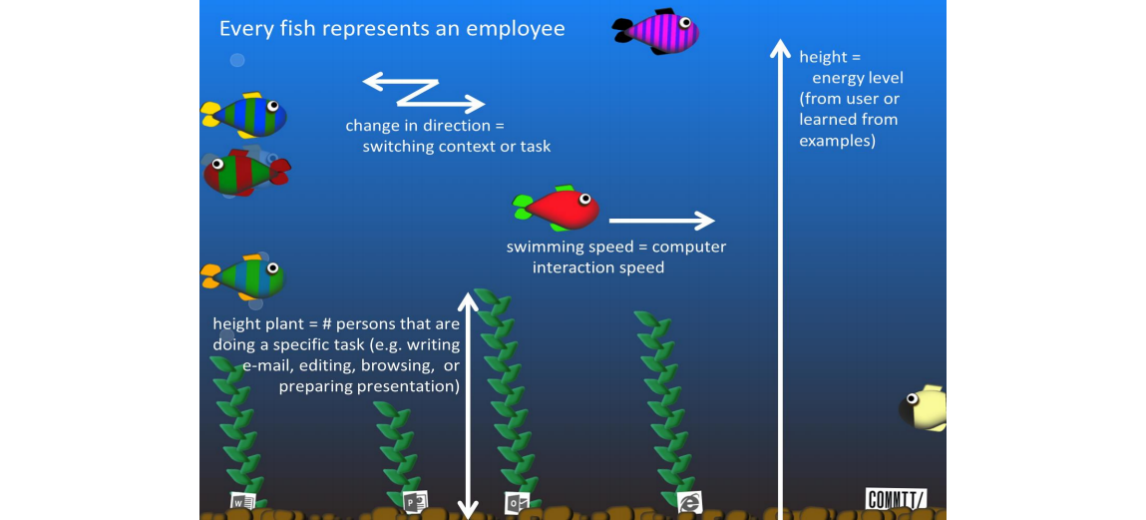
Screen image of the SWELL Fishualization placed in the coffee corner.

The SWELL Fishualization (for details we refer to the original work presented in [51] and [52]) is aimed at enabling employees to gain insights into their working habits and encourage social interaction about healthy working, in order to improve well-being at work (T05). It provides a feedback screen in the form of a digital fish tank (Figure 11), which is placed at a central location in the office. The primary sensor is currently a key-logging software that is installed on the user’s computers. Other sensors could also be coupled to add information on, heart rate, dominant facial expression, or e-mail sentiments. Each fish in the Fishualization represents an individual employee. The speed of a fish is determined by how fast the corresponding employee is interacting with their computer (number of clicks and keystrokes) and the number of changes in direction represents the number of task or context switches. The y position of each fish currently represents the (self-reported) energy level of the corresponding employee. Plants at the bottom of the screen represent performed tasks, for example writing e-mails, editing documents, browsing, or preparing presentations. The more people worked on a tasks the larger the plant.

SWELL Fishualization tries to tackle stress in the middle of the stress chain by helping employees to cope with stress (secondary prevention). It is aimed at enhancing support from colleagues, thus addressing the working conditions. Its main basis is the JD-R model (providing additional resources). It measures work characteristics and assesses the energy dimension of engagement by means of user input. With respect to behavioral change it helps with monitoring the current situation. Moreover, it fosters the motivation to take action by means of a playful approach and social influences.

We evaluated the prototype in a real-world environment. The Fishualization trial at the Media and Network Services group at TNO (Dutch institute for applied scientific research) ran for about 2.5 months (March to May 2014). The Fishualization screen (a large computer display) was placed in the coffee corner. A subset of 10 employees volunteered to couple their computer interactions and subjective input of their energy level to one of the fish. In order to measure the effects of the deployment of the Fishualization, all employees who use the coffee corner were asked to fill in pre- and post-questionnaires on personal awareness of working patterns and well-being at work, group awareness, and interactions with colleagues. Furthermore, camera and microphone recordings were used to measure activity at the coffee corner. To ensure privacy, only the number of detected faces, the amount of video motion, and the average sound level were deduced and stored (no video or sound was stored). This data collection started 3 weeks before the Fishualization was turned on and continued during the trial to compare activity in the coffee corner before and after deployment of the Fishualization.

In all, 30 employees filled in the pre-questionnaire and 14 employees filled in the post-questionnaire. The subset of respondents did not differ significantly in their current level of well-being or how content they were about their well-being. We used independent samples *t* tests to compare the pre- and post-test results. A significant effect on the item “I am aware of typical patterns in working behavior throughout the day or week (eg, mailbox on Monday morning, project work after lunch...)” was found (*P*=0.004). Awareness of working patterns was higher in the post-test than in the pre-test with mean (SDs) of 4.79 (1.626) and 3.27 (1.530), respectively (scale 1 “not” to 7 “very much”). Moreover, we found a significant effect on the item “I know how I can change my working behavior to gain a better level of well-being (eg, becoming more productive, reducing stress...)” (*P*=0.005). The mean (SD) score in the post-test 5.14 (1.231) was higher than in the pre-test 3.9 (1.322).

We conclude that the Fishualization caused more personal awareness on working behavior and its relation with well-being among employees. However, we did not find significant effects on items related to group awareness and interactions with colleagues. In the further development of the Fishualization we should focus on fostering social interaction among colleagues more (eg, by adding new functionality), as this may be a good buffer against stress. Moreover, most participants were enthusiastic about the Fishualization: a playful manner of feedback turned out to be engaging. Finally, we used sensor technology to quantify activity in the coffee corner, which shows the potential of new technology for experimental evaluation.

#### Providing Tips - SWELL NiceWork E-Coach

**Figure 12:**
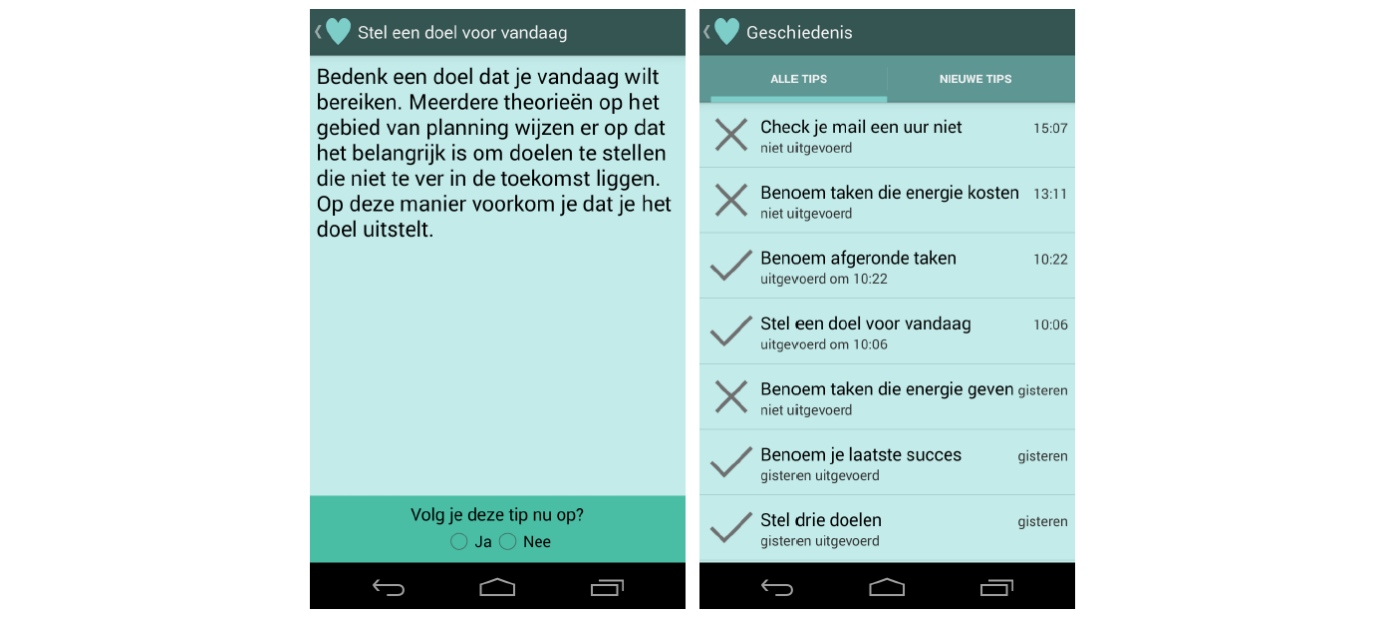
The SWELL NiceWork app.

The SWELL NiceWork app (for details we refer to the original work presented in [53]) is designed to provide coaching for short recovery breaks (T07). The app provides simple tips, three times a day, aimed at promoting well-being at work (Figure 12). Various scientific articles, websites, and magazines on well-being at work were reviewed to collect appropriate tips which resulted in a list of 54 tips. Each tip does not take more than three minutes, and no special materials or specific locations are required. The recommended well-being tips are cognitive-behavioral, creative, physical exercises, food, journaling, relaxing, social, and time-management.

We found that different people had different preferences for tips (pilot study, in which 26 employees rated their preferences for the 54 tips). Therefore, a recommendation approach was chosen to adapt which tips are given to the specific user. After each recommendation, the user can indicate whether he performed the tip and the system learns over time to give better tips.

The SWELL NiceWork e-coach is mainly aimed at supporting the work-rest balance. The app also provides tips aimed at preventing the experience of stress (secondary prevention), as tips on recovery from coping with high demands (tertiary prevention). The tips focus on personal factors or the working context. Its main basis is the E-R model (focusing on recovery). It does not yet measure anything. The system does assess whether the user has followed-up a tip by means of user input. With respect to behavioral change it helps with taking action and learning new behavior by providing triggers and suggestions.

To evaluate the NiceWork app with users, 35 employees tested the e-coach for 2 weeks. The first hypothesis was that knowledge workers have a positive attitude towards the e-coach. This hypothesis was confirmed in the user study. The number of followed-up tips was high (2 out of 3 per day) and most participants agreed that it is pleasant to receive automatic notifications. The study also showed that three recommendations per day seemed a right amount of suggestions. Moreover, indicating whether a tip was followed-up and asking for a short motivation when a tip was rejected turned out to be a well-designed method for providing feedback. Our second hypothesis was that tailored recommendations are followed-up more often compared to randomized suggestions. We did not find strong evidence for this hypothesis. Results show that our recommendation method, which provides tailored suggestions, did not substantially increase the number of tips that were performed compared to a method that provided randomized suggestions.

Furthermore, results show that of all tips that were not followed-up, only a few were rejected because it was disliked (12.8%, 45/350). Instead, tips were mostly rejected because the moment of recommendation was somehow inappropriate including wrong timing (46.0%, 161/350), not relevant (14.8%, 52/350), or not at work (14.0%, 49/350). This finding suggests that future e-coaches may increase their effectiveness by recommending tips at appropriate times. Using sensor information to ensure that tips are suggested just-in-time was the most important personalization method that needed to be further explored [54]. Moreover, we demonstrated that technology can be used to investigate the effects of an intervention: via the app we directly investigated how many interventions were said to be followed-up and we directly asked for reasons for not following up a suggestion.

#### The Evaluation of Prototypes

Here, we presented the general SWELL functionality and described two prototypes and their evaluation. Within the SWELL project, however, other prototypes were developed and evaluated [55].

In general we can say that we made working implementations of some pervasive technologies for improving well-being at work. Our evaluation until now was mainly aimed at user experience and testing underlying technologies. The evaluation yielded several additional requirements for our system. Moreover, we showed how technology can be used to investigate the effects of an intervention. In further research we should also evaluate whether the prototypes have the expected positive effect on employee’s well-being at work. From our small scale pilot studies we got some first insights, but ideally the systems are evaluated with in a much larger field test.

## Discussion

### Principal Findings

By means of situated cognitive engineering [10], we combined stress and intervention theory with knowledge of technological possibilities and input by users to design a pervasive system that helps knowledge workers to improve well-being at work. The questions answered in this paper are discussed in Textbox 4.

**The questions addressed in this paper and their relevant discussions.**

Questions

1. Which concepts are relevant with respect to well-being at work? We found that the relationship that people have with their jobs can be described as a continuum between engagement and burn-out [12]. Engagement is characterized by energy, involvement, and efficacy or absorption. Biology describes more short term effects of stress [3]. A stressor causes a particular perception of the stressor in the individual. This can lead to acute physiological stress responses and, in the long run (due to lack of recovery) long-term physical, cognitive, emotional, and behavioral stress consequences.

2. Which person, work, and context conditions can lead to negative stress? There are no specific personal, work, or context conditions that generally lead to stress. Work becomes stressful when high demands are combined with insufficient resources, little rewards, little recovery, or an environment that mismatches with personal characteristics. The most useful models for developing technology based interventions are the JD-R model [21] and the E-R model [26]. We presented several ideas to diminish demands, enhance resources, or help with recovery.

3. How can sensors be applied to automatically infer stress and the context in which it appears? We can use technology to sense work characteristics (eg, tasks and topics worked on), measure acute physiological stress responses in the body (eg, HRV), or assess cognitive, emotional and behavioral effects of stress (eg, sleep duration). The user study showed that users are only interested to collect data that is necessary for supporting their specific goal, so the system should be configurable.

4. Which interventions can be provided by means of pervasive technology to help a knowledge worker improve his well-being at work? In general, three stress prevention approaches are distinguished, aimed at different stages in the stress chain [45]. Technology can thus either address the stressor (eg, by providing work support), address short-term stress reactions (eg, by enhancing coping), or address long-term stress consequences (eg, helping to improve recovery). Suitable behavioral change techniques [49] should be used to address the motivation, ability, or trigger to take action (eg, self-monitoring and reminders to action).

We presented the resulting general framework in which we related several relevant theories. We related work on engagement and burn-out [12] to work on stress [3], and described how relevant aspects can be quantified by means of sensors. We also outlined underlying causes of work stress [21,26], and described how interventions can address these [45], in particular by means of new technologies utilizing behavioral change theory [49]. This framework can be used by other researchers to design pervasive systems that address well-being at work.

Finally, we described the envisioned SWELL system and core functionality that was identified. We also presented two built prototypes: the SWELL Fishualization [51,52] that provides department wide feedback for peer support, designed to improve coping, and the SWELL NiceWork e-coach [53] that provides well-being tips, designed to improve coping or recovery. All in all, we demonstrated the (technological) feasibility of our ideas. First evaluations with users were positive and provided further insights to refine the systems.

### Limitations

The biggest challenge in developing our comprehensive and practical framework was the vast amount of available concepts and models regarding well-being at work. We consulted experts in the field and had to make choices on what concepts and theories to include. Our selection may reflect our specific scoping, such as addressing work stress in the population of knowledge workers. We focused on providing a general and simple overview, combining different areas of research.

Another challenge in this respect was relating concepts of different fields to each other. These concepts differ in their level of abstraction: organizational psychology provides the most high-level terms, including the relation between resources versus demands or recovery [12]. Biological theories provide more low-level terms, such as physiological stress responses in the body [3]. Our aim was to make several of these aspects quantifiable. This means translating these concepts into even more low-level terms, such as a specific sensor, the data to be collected, and the interpretation of this data.

Besides the high-level versus low-level continuum, there is also a temporal continuum from short-term stress [3] to developing a burn-out [12]. In traditional approaches with questionnaires, mainly long-term aspects are assessed. Sensing, however, enables real-time measurements in real-world work settings. We aimed to translate relevant aspects identified based on theories into variables that are measurable at the workplace.

The resulting general and pragmatic framework provides a structure to develop pervasive technology for improving well-being at work. We noticed that far more diverse technology-based interventions can be developed than initially assumed. The theoretical foundation gave many different pointers of how well-being at work can be improved from coaching during work, over fostering social support, to addressing recovery after work. Besides the ideas and prototypes presented here, many more (technological) solutions can be developed based upon this general framework (eg, teaching coping in an online course, building a social network for peer support, enhancing recovery by letting people play a computer game).

We built prototypes of some pervasive technologies for improving well-being at work [51-53]. Our evaluation until now was mainly aimed at user experience and testing underlying technologies. Further research should evaluate whether the prototypes have the expected positive effect on employee’s well-being at work. From our small scale pilot studies we got some first insights, but ideally the systems are evaluated with in a much larger field test.

As a final note, we need to be cautious to put responsibility for managing work stress at the individual level. Certainly the company and management also play a role. Therefore, an intervention provided one-on-one at an individual by means of a pervasive system is ideally part of a larger intervention program. In case many employees struggle with similar problems, a department wide intervention may be more effective. Furthermore, specific problems at work may not be solvable by the employee himself. In this case, the management or organization may need to be approached.

### Reflection

We think a pervasive system aimed at an individual’s abilities to cope with stress and improve well-being at work poses many new opportunities. A system using real-time during work can provide much valuable information on work stress. Moreover, employees can be empowered to self-manage their well-being at work by means of tailored interventions. Throughout our work we encountered several challenges and opportunities for further research in several categories (Textbox 5).

**Challenges and opportunities for further research.**

Opportunities and challenges

1. Multi-disciplinary, theory and data-driven research, and development. New technology brings new possibilities. The now very abstract models can be more refined to include directly measurable concepts and new types of support. New technology can also be used to directly evaluate the success of an intervention. Sensors can be used to investigate how far interventions are indeed followed-up (eg, whether users take a break or become physically active after a suggestion by an e-coach). Moreover, the effects of an intervention can be measured (eg, whether provided information support indeed decreased mental effort and stress). Technical experts and social scientists should aim to work together. It is therefore necessary that the experts understand each others' domains well, which is challenging.

2. Interpreting personal sensor data. Sensor data is relatively easy to collect, the challenge is making sense of this data. We should investigate which behavior is indicative of stress during work and how these can best be captured by means of unobtrusive sensors. People differ in their (work) behavior, so there is a need to build personalized models This brings methodological challenges, such as how to instantiate a model for a new user.

3. Relation between measurable aspects and burn-out. In future work, the relation between subjective experience based upon our own feelings and objective measures based on objective data should be investigated. Can objective measurements help us with detecting stress? Ideally, a system would be able to give a warning in case it predicts that the current behavioral pattern will cause long-term problems. Therefore, research should be done on how longitudinal patterns in sensor data relate to long-term stress consequences and burn-out.

4. Combining strengths of human and technology. Ideally, the strengths of technology (eg, being objective or persistent) and the strengths of a human (eg, being good in interpretation) should be combined. The role of the system and the user should be clear. The most suitable manner for pervasive technology to interact with an employee is a challenging question for human-computer interaction research. Issues of control are important. The system needs to interact in a way that provides support, while not irritating the user.

5. Privacy. The success of pervasive systems collecting context data depends on the acceptance by users. A system that collects personal data raises many privacy questions. Therefore, privacy should be integral part of the design process (eg, doing a Privacy Impact Assessment or implementing Privacy by Design).

6. Ethics. Measuring and trying to change the behavior of individuals poses all kinds of ethical questions. Is it acceptable to monitor and change the behavior of an employee? It is difficult to predict how such new pervasive e-coaching systems will be perceived and used (or even misused) when applied in real-world work settings.

### Conclusions

In this work we developed a theoretical framework for the design of pervasive well-being technology. The framework is based on several relevant work stress and intervention theories, as well as possibilities that new technologies as sensors and mobile phones provide. This framework can be used to systematically develop theory-based technology supported interventions to address work stress, as we demonstrated in our SWELL case study.

## Abbreviations

AWS: Areas of Worklife Scale
e-coach: electronic coach
E-R model: Effort-Recovery model
ERI: Effort-Reward Imbalance model
GPS: Global Positioning System
JD-R model: Job Demands-Resources model
P-E fit model: Person-Environment fit model
SWELL: Smart Reasoning for Well-being at Home and at Work project
